# Survival Following Steroid-Based Therapy in a Case of Penicillin-Induced Stevens-Johnson Syndrome and Toxic Epidermal Necrolysis Overlap

**DOI:** 10.7759/cureus.85396

**Published:** 2025-06-05

**Authors:** Virgilio Blandon, Miguel Borge

**Affiliations:** 1 Department of Dermatology, Hospital Carlos Roberto Huembes, Managua, NIC

**Keywords:** dexamethasone, drug eruptions, drug hypersensitivity, glucocorticoids, immunomodulation, penicillin, scorten, stevens-johnson syndrome, stevens-johnson syndrome/toxic epidermal necrolysis overlap, toxic epidermal necrolysis

## Abstract

Stevens-Johnson syndrome (SJS) and toxic epidermal necrolysis (TEN) are life-threatening mucocutaneous reactions with mortality rates strongly correlated with disease severity. We report the case of a 47-year-old Mestizo man in Nicaragua with penicillin-induced SJS/TEN overlap syndrome (body surface area involvement: 10%-30%, score of toxic epidermal necrolysis, SCORTEN: 4, and predicted mortality: 58%). The patient developed mucosal erosions, hemorrhagic crusting, and disseminated erythematous plaques following exposure to amoxicillin and dicloxacillin (penicillin-class antibiotics). Initial misdiagnoses delayed care, but hospitalization prompted early intravenous dexamethasone, fluid resuscitation, topical corticosteroids, and immediate discontinuation of the offending agents. Epidermal detachment halted within 72 hours, with complete reepithelialization by day 10. Transient hyperglycemia resolved spontaneously, and the patient survived without infections or sequelae, contrasting with the SCORTEN-predicted mortality. This outcome supports early immunomodulation to mitigate cytokine-driven necroptosis, challenging historical concerns about corticosteroid risks. Limitations include the single-case design, absence of histopathology, and lack of human leukocyte antigen allele screening for pharmacogenomic insights. The case underscores the efficacy of prompt corticosteroids in high-risk SJS/TEN linked to penicillin-class drugs, emphasizing drug-specific vigilance and discontinuation. It highlights the need for population-specific SCORTEN calibration, pharmacogenomic integration, and publication of rare cases to enhance regional epidemiological understanding.

## Introduction

Stevens-Johnson syndrome (SJS), toxic epidermal necrolysis (TEN), and their overlap (SJS/TEN) are life-threatening conditions where the skin and mucous membranes (like those in the mouth, eyes, and genitals) are severely affected. Characterized by extensive epidermal detachment, meaning the top layer of the skin essentially separates from the layers beneath, much like a severe burn, and significant mucosal involvement, these are acute medical emergencies. They are classified by the percentage of body surface area (BSA) detachment: under 10% for SJS, 10%-30% for SJS/TEN overlap, and over 30% for TEN. Drug reactions are the primary cause, triggering the body's immune system to attack its own skin cells [1].

Prognosis for SJS/TEN is assessed using the score of the toxic epidermal necrolysis (SCORTEN) system, the gold standard for predicting mortality. This tool considers variables such as age, presence of malignancy, heart rate (HR), extent of BSA detachment, and certain laboratory values, including serum urea, bicarbonate, and blood glucose levels [2]. SCORTEN is widely used to evaluate patient outcomes and compare treatment effectiveness across different settings [3-6]. Medical guidelines, including those from the UK, recommend calculating the SCORTEN score upon admission to help guide clinical decisions and risk stratification [7]. However, its accuracy can vary in distinct ethnic populations and specific patient groups, as some studies suggest it may overestimate or underestimate mortality risk depending on the cohort, underscoring the need for context-specific validation [8,9].

The incidence of SJS and TEN varies globally. In the United States, for example, the estimated annual incidence is 9.2, 1.6, and 1.9 cases per million adults for SJS, SJS/TEN overlap, and TEN, respectively [10]. Notably, certain non-White populations, particularly Asians and Blacks, face a disproportionately higher risk [10,11]. This elevated risk often correlates with specific genetic predispositions, known as human leukocyte antigen (HLA) alleles. These HLA proteins help the immune system distinguish between the body's own cells and foreign substances. However, certain variations in these HLA genes can cause the immune system to mistakenly recognize a drug as a threat, leading to a severe reaction against the body's own skin cells [11].

Etiologically, antibiotics, anticonvulsants, and analgesics are the drug classes most frequently implicated in SJS/TEN; however, nondrug triggers, such as *Mycoplasma pneumoniae* infections, can also occur [12,13]. Coexisting medical conditions, including cancer, HIV, and autoimmune diseases, can worsen the patient's prognosis [12].

Management of SJS/TEN involves promptly stopping the causative drug, providing meticulous supportive care, and often using immunomodulatory therapies [6,7]. While the use of systemic corticosteroids in SJS/TEN remains a topic of debate, some studies suggest benefits [4,5,14,15]. In contrast, others report no clear mortality advantage [6,14], highlighting the ongoing need for more research [16].

This report presents the case of a patient with SJS/TEN overlap syndrome (SCORTEN: 4, predicted mortality: 58%) who survived after receiving a steroid-based treatment. This outcome emphasizes the complexities of predicting mortality and the potential effectiveness of early immunomodulation, contributing to the ongoing discussion on optimizing care for this critical condition.

## Case presentation

A 47-year-old Nicaraguan man, weighing 106.5 kg, with class III obesity (body mass index: 37.96 kg/m²) and recently diagnosed type 2 diabetes mellitus, initially presented with pruritic urticarial lesions on his knees and odynophagia. On day 0, he began treatment with oral amoxicillin and prednisone 50 mg daily. On day 2, during a follow-up consultation, with his lesions misinterpreted as bacterial superinfection, dicloxacillin was prescribed, and prednisone was discontinued.

**Figure 1:**
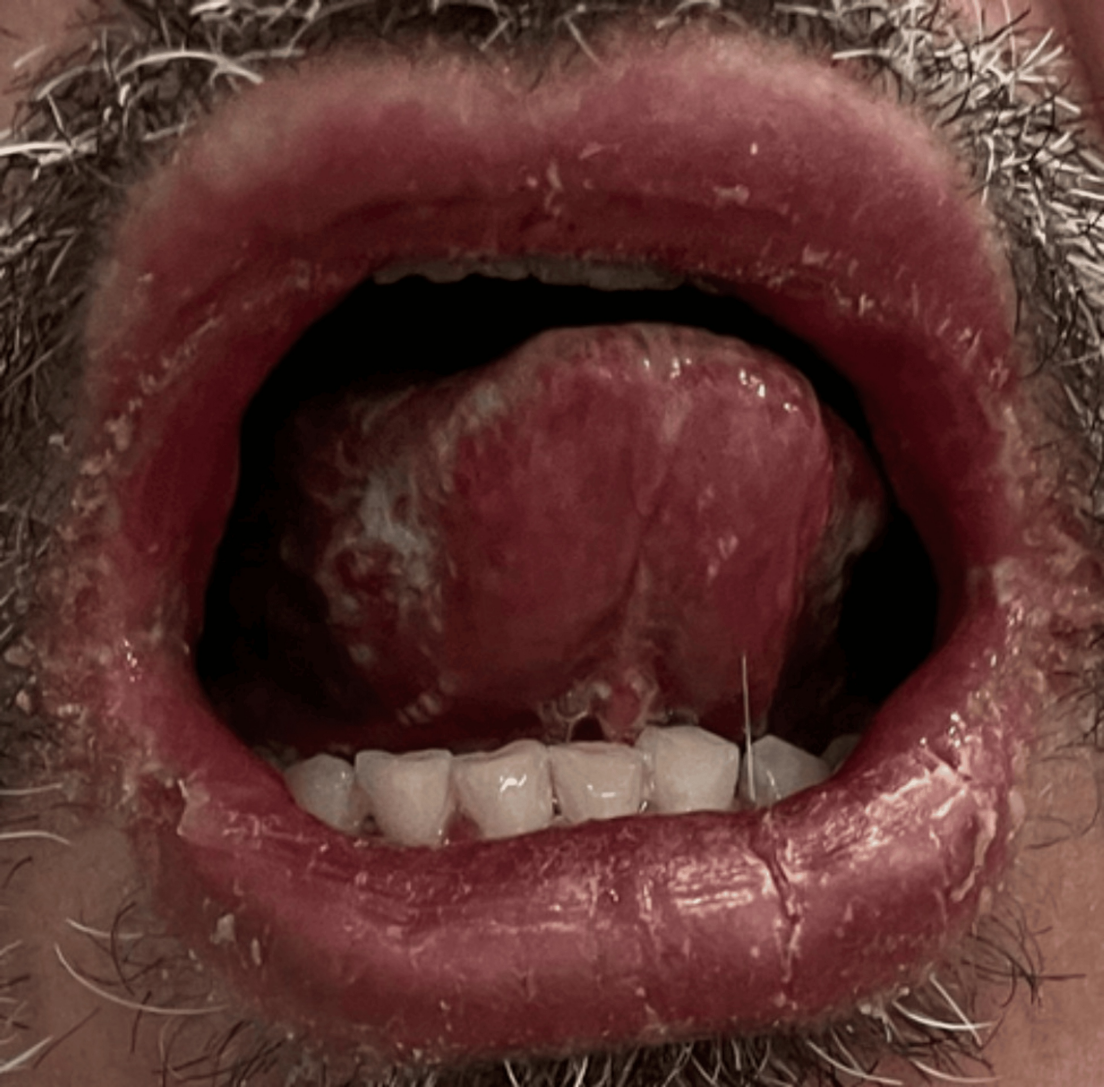
Mucosal involvement in SJS/TEN Erythema and erosions are observed on the oral mucosa with serohemorrhagic crusting on the red border of the lips SJS: Stevens-Johnson syndrome; TEN: toxic epidermal necrolysis

**Figure 2:**
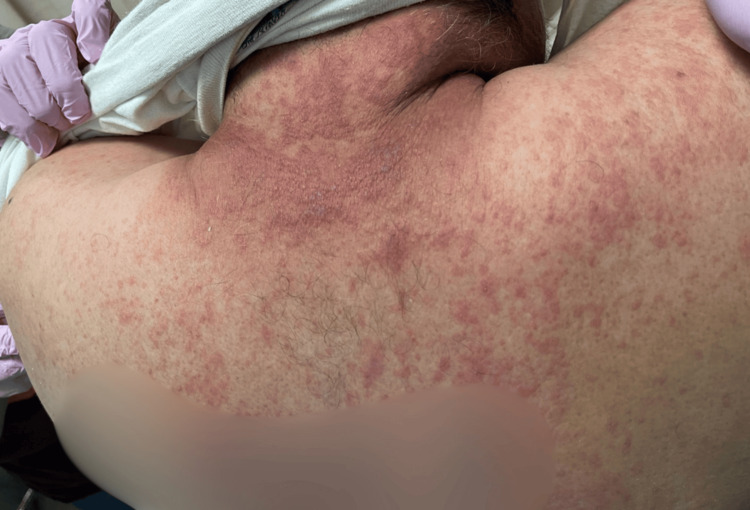
Erythematous macules, some isolated on the upper back, which are confluent, and small blisters are evident on their surface

**Figure 3:**
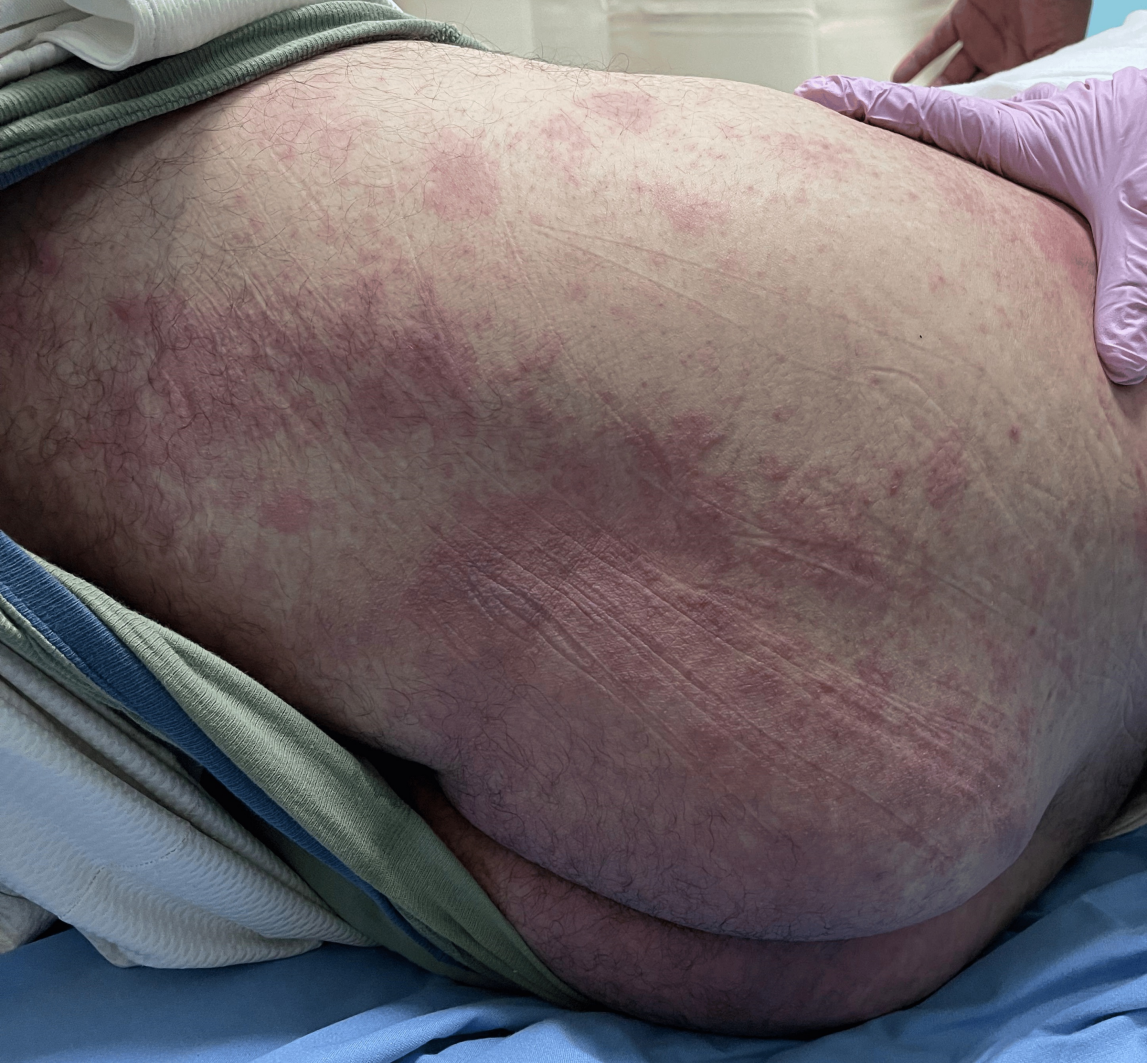
Erythematous macules, some isolated, affecting parts of both buttocks, are confluent and present small blisters on their surface

He took dicloxacillin for four days. During this period, as reported by the patient upon admission, he developed painful oral ulcers, hemorrhagic lip crusting (Figure 1), conjunctival erythema, and rapidly spreading vesiculobullous lesions on his trunk and extremities (Figures 2, 3). The dicloxacillin exacerbated these lesions.

On day 6, he presented to the emergency department with fever (40°C) and malaise, but no other specific systemic symptoms. At this time, the epidermal detachment was calculated at approximately 30% of his BSA, and he was admitted with a diagnosis of SJS/TEN overlap syndrome. The patient had a SCORTEN score of 4 upon admission, corresponding to a predicted mortality of approximately 58% (Table 1) [2].

**Table 1:** SCORTEN criteria (toxic epidermal necrosis score) SCORTEN predicts mortality in SJS/TEN: 0-1: <10%, 2: ~12%, 3: ~35%; 4: ~58%, ≥5: >90% SCORTEN: severity of illness score for toxic epidermal necrolysis; SJS: Stevens-Johnson syndrome; TEN: toxic epidermal necrolysis

SCORTEN criteria	Point
Age >40 years	1 point
Presence of malignancy (active cancer)	0 point
Heart rate >120 beats/minute	0 point
Body surface area detached >10%	1 point
Serum urea >10 mmol/L (≈28 mg/dL)	0 point
Serum glucose >14 mmol/L (≈250 mg/dL)	1 point
Serum bicarbonate <20 mmol/L	1 point
Total points	4 points

**Figure 4:**
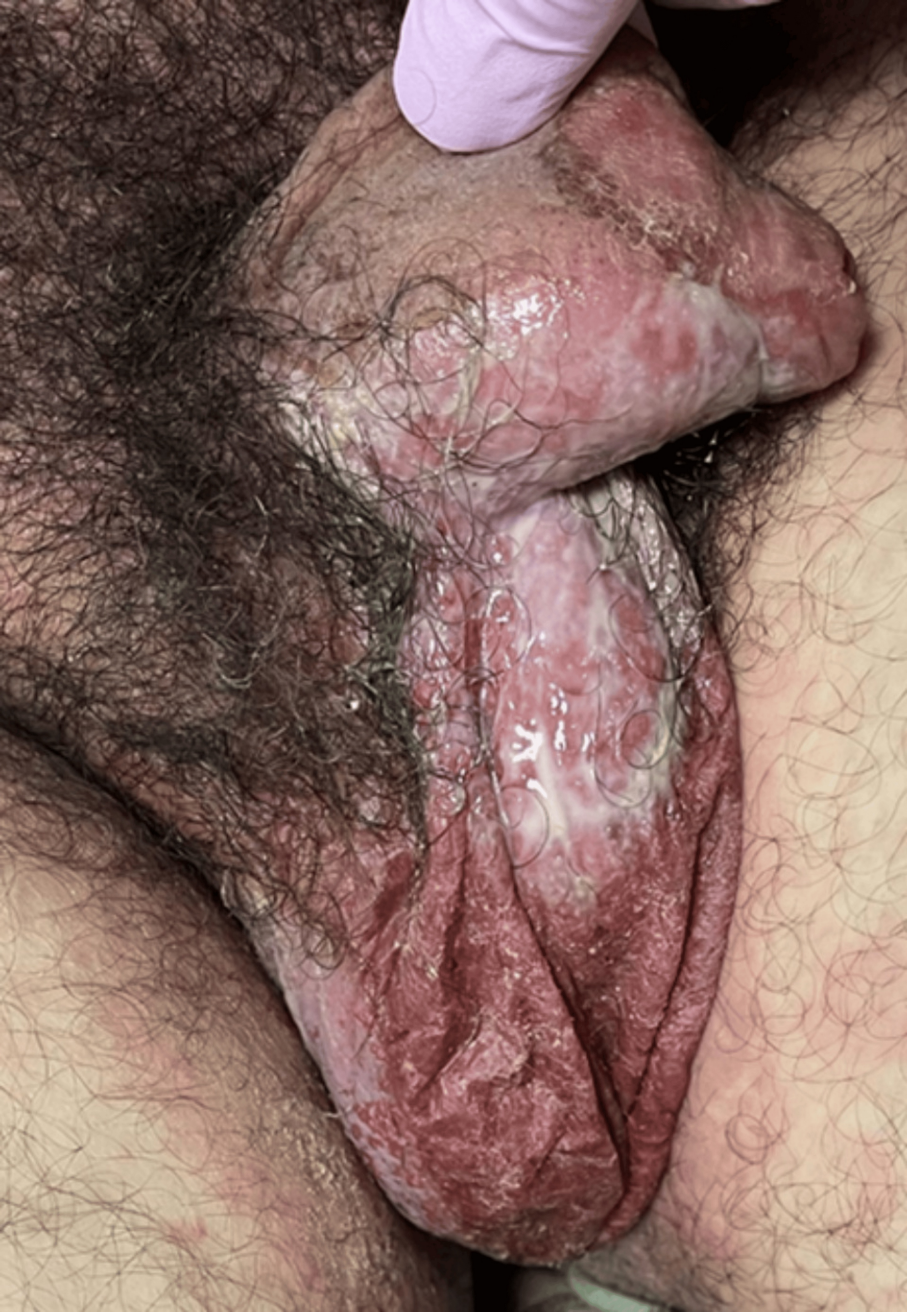
Mucosal involvement in SJS/TEN, where erosions are observed in the genital mucosa, affecting the scrotum and penis SJS: Stevens-Johnson syndrome; TEN: toxic epidermal necrolysis

On admission, he was hemodynamically stable (BP: 120/70 mmHg, HR: 84 bpm). Dermatological examination revealed extensive erythema and widespread vesiculobullous lesions (Figures 2, 3) that included flaccid bullae, with Nikolsky-positive areas, primarily distributed on the posterior trunk, buttocks, posterior thighs, and anterior trunk. Mucosal involvement included oral erosions, genital ulcerations (Figures 1, 4), and bilateral conjunctival injection without corneal damage.

**Figure 5:**
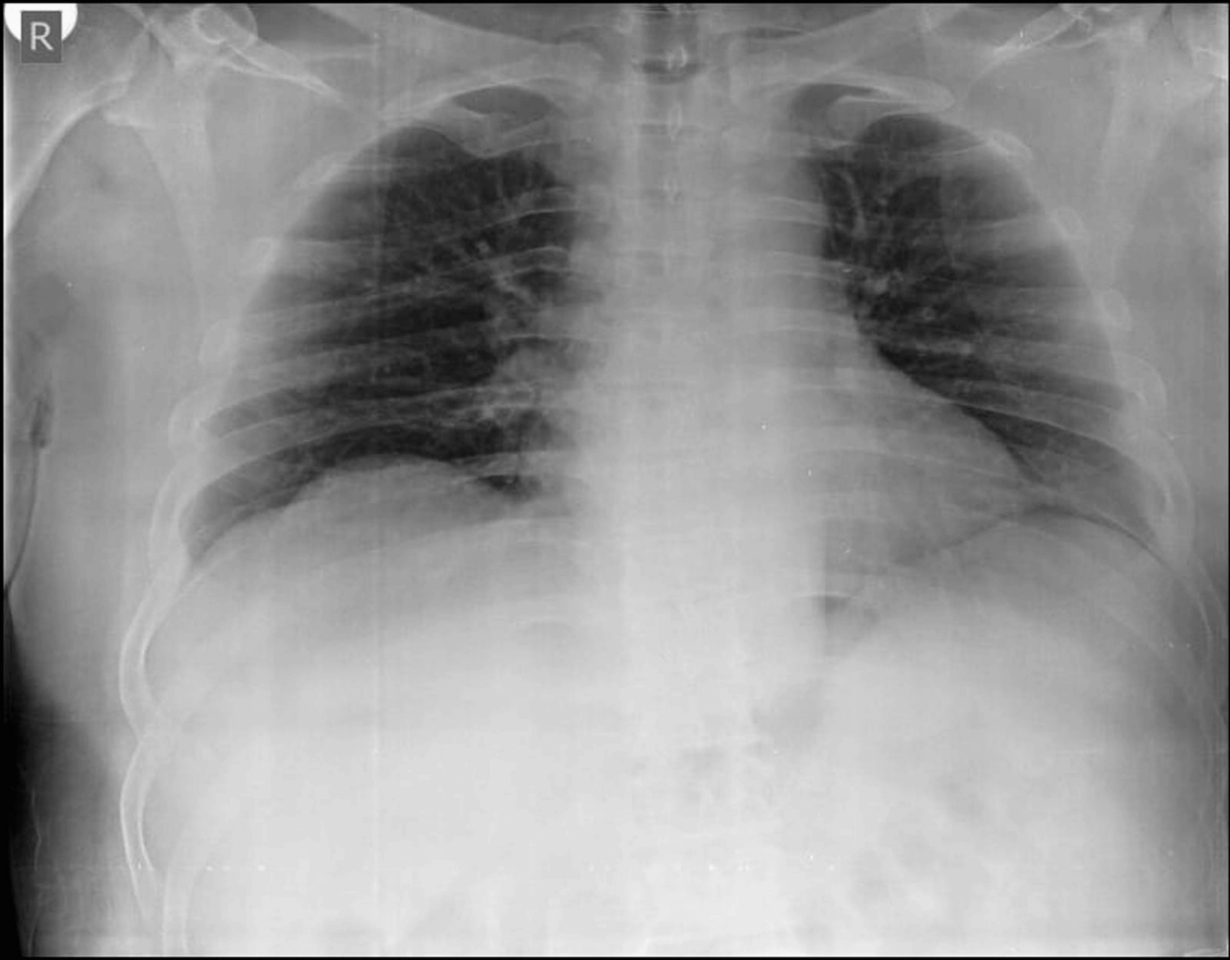
Chest X-ray Posteroanterior projection. In the lower left region, there is a reinforcement of the vascular network and an increase in density suggestive of interstitial infiltrates. The radiologist ruled out pneumonia; findings likely reflect noninfectious pulmonary changes related to Stevens-Johnson syndrome/toxic epidermal necrolysis overlap

A chest X-ray (Figure 5) ruled out atypical pneumonia (*M. pneumoniae*), supporting a drug-induced etiology. Laboratory findings (Table 2) included leukocytosis (WBC 15.8 × 10³/µL), hyperglycemia (275 mg/dL), and serum bicarbonate of 18 mmol/L. Serologies for HIV, hepatitis B/C, and syphilis were negative.

**Table 2:** Laboratory data Includes data from complete blood count, blood chemistry, and arterial gasometry Reference values used correspond to intrahospital laboratory standards for adult patients hospitalized at our institution The mild elevations in GGT and ALT (≈6 IU) are likely related to systemic inflammation from the drug reaction and are not considered predictive or clinically significant findings ^*^Measured in microliters ^**^Value that is both abnormal and included among the SCORTEN prognostic criteria (e.g., serum glucose >250 mg/dL and bicarbonate <20 mmol/L) HCO_3_: bicarbonate ion; GGT: gamma glutamyl transferase; ALT: alanine transaminase; SCORTEN: severity of illness score for toxic epidermal necrolysis

Exam	Result	Reference values
Lymphocytes	3.20 × 10^3^/uL^*^	0.8-4 × 10^3^/uL
Segmented	67%	50%-70%
Platelets	231 × 10^3^/uL^*^	150-450 × 10^3^/uL
Chemistry		
Serum creatinine	0.72 mg/dL	0.4-1.4 mg/dL
Glucose (blood sugars)	275 mg/dL^**^	70-105 mg/dL
Blood albumin	3.4 g/dL	3.5-5.3 g/dL
Albumin-to-globulin ratio	0.99	1.2-2.2
Aspartate aminotransferase	16.8 U/L^*^	0-32 U/L
Alanine aminotransferase	51.7 U/L^*^	0-45 U/L
Alkaline phosphatase	75 U/L^*^	30-120 U/L
GGT	68.6 U/L^*^	9-61 U/L
Urea nitrogen	5.4 mg/dL	4.6-23.4 mg/dL
Urea	11.6 mg/dL	10.1-50.1 mg/dL
Arterial blood gas analysis		
HCO_3_	18 mmol/L	22-32 mmol/L
Serology		
Hepatitis C virus antibodies rapid test	Negative	-
Hepatitis B virus surface antigen	Negative	-

Skin biopsy was deferred due to prior corticosteroid use (prednisone 50 mg daily initiated at first consultation), which may confound histopathological interpretation, and the unequivocal clinical presentation aligning with SJS/TEN overlap. The diagnosis was confirmed by 30% BSA involvement (overlap criteria: 10%-30%) and temporal association with amoxicillin/dicloxacillin. During the initial assessment, the patient reported a history of a similar, milder allergic reaction (described as urticaria) associated with benzathine penicillin less than two years prior. The causality between the medications and the reaction was assessed using the algorithm of drug causality in epidermal necrolysis (ALDEN) score [17], yielding a score of 7 points, which indicates "very probable" drug causality for amoxicillin/dicloxacillin (Table 3).

**Table 3:** ALDEN score ALDEN is a validated tool to assess the likelihood that a drug caused SJS/TEN A total score of 6 or more indicates a very probable causal relationship between the drug and the reaction. A score of 4-5 suggests a probable association, while 2-3 is considered possible. A score of 0-1 makes the association unlikely, and a score of less than 0 indicates a very unlikely link between the drug and the adverse reaction ALDEN: algorithm of drug causality in epidermal necrolysis; ED: emergency department; SJS: Stevens-Johnson syndrome; TEN: toxic epidermal necrolysis

Criterion	Patient case description	Score
I. Delay from initial drug intake to onset of reaction	Dicloxacillin was started on day 2; symptoms rapidly progressed over the four days of use, with ED presentation on day 6. This implies onset within one to four days after new exposure	3
II. Drug present in the body on index day	Dicloxacillin was taken up to the day of ED presentation (day 6)	0
III. Prechallenge/rechallenge (history of similar reaction)	Patient reported a milder allergic reaction (urticaria) to benzathine penicillin less than two years ago (a similar drug)	1
IV. Dechallenge (improvement after withdrawal)	All antibiotics were discontinued upon admission. Epidermal detachment stopped within 72 hours, reepithelialization began by day 7, and full mucosal healing occurred by day 14	0
V. Type of drug (notoriety)	Penicillins (e.g., amoxicillin and dicloxacillin) are strongly associated with SJS/TEN according to previous case-control studies	3
VI. Other causes	Chest X-ray ruled out *Mycoplasma pneumoniae*; serologies for HIV, hepatitis B/C, and syphilis were negative. No other drug with a higher intermediate score was implicated	0
Total ALDEN score	-	7
Interpretation	-	Very probable

**Figure 6:**
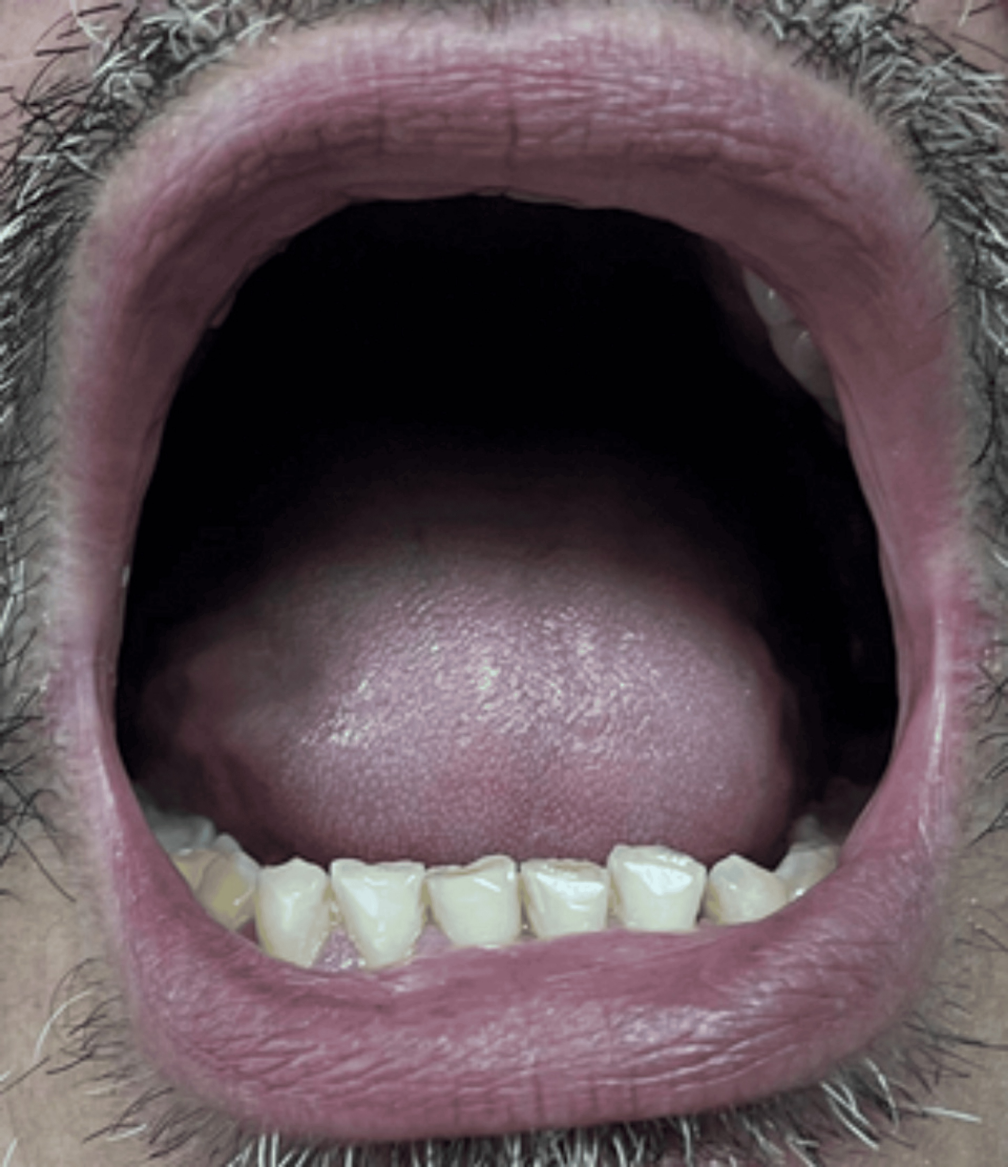
Oral mucosa (lips), where no erythematous lesions are evident

**Figure 7:**
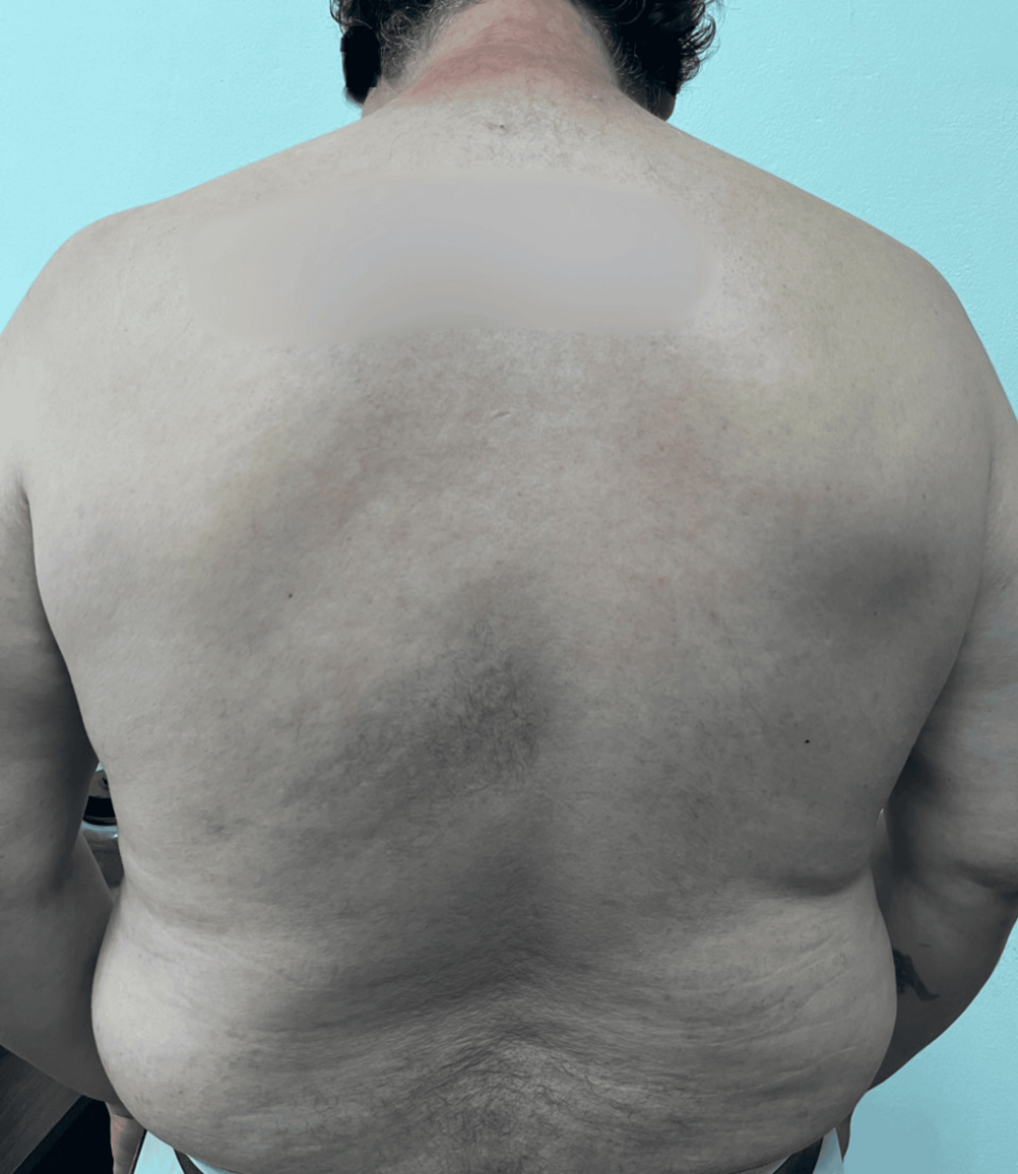
Back region showing no evident erythematous lesions

**Figure 8:**
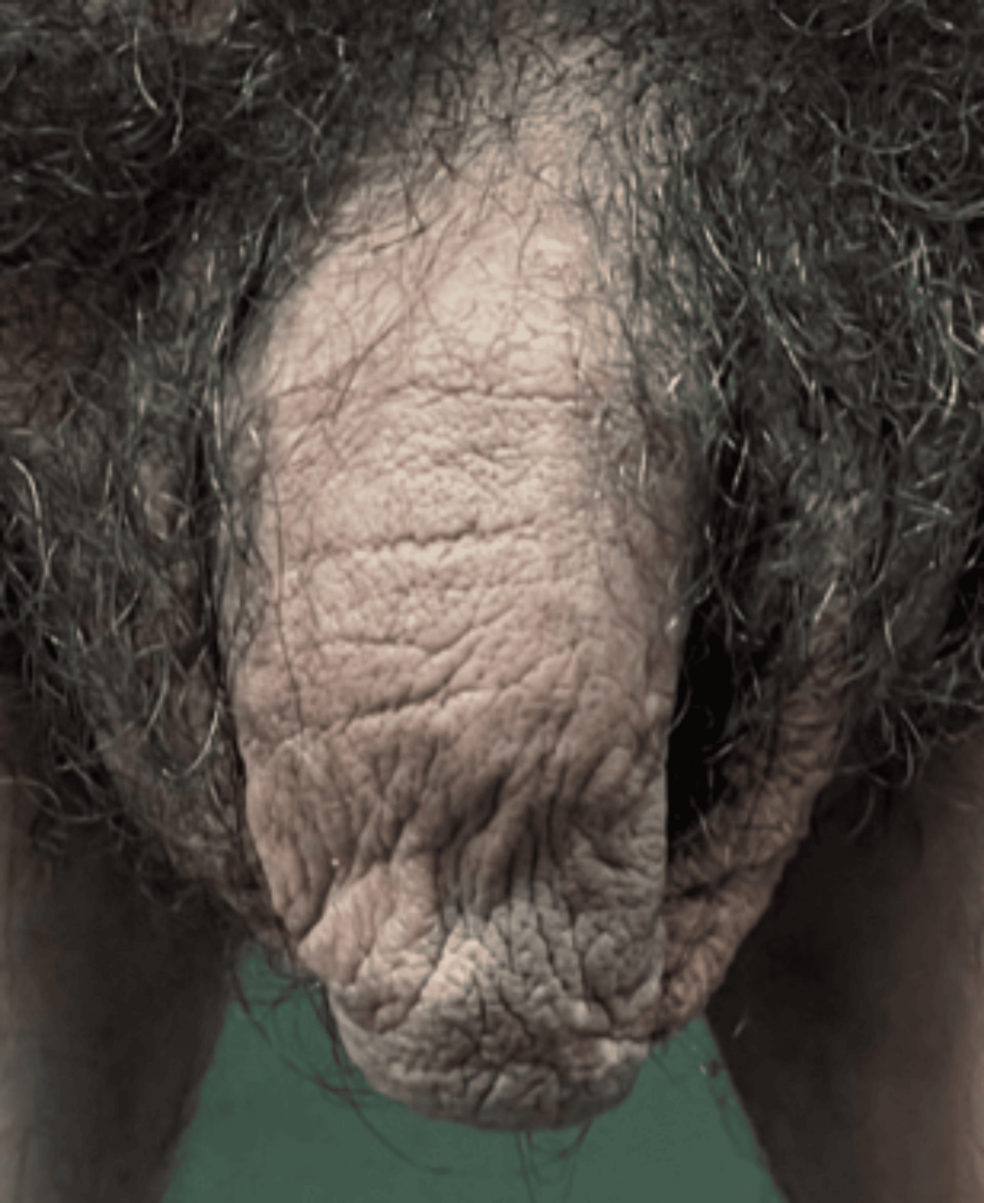
Genital region showing no evident erythematous lesions

All antibiotics were discontinued. Balanced fluid therapy with Hartmann's solution (750 mL IV every 12 hours) was initiated to address insensible losses while avoiding volume overload. Urine output was closely monitored via a Foley catheter, maintaining a target diuresis >0.5 mL/kg/h to prevent acute kidney injury. Moderate-dose dexamethasone (0.15 mg/kg/day) was administered for seven days, followed by an oral prednisone taper. Supportive care included topical betamethasone 0.1% paste and zinc oxide for erosions, saline mouth rinses, insulin for glycemic control, and thromboprophylaxis with heparin. Epidermal detachment halted within 72 hours, reepithelialization began by day 7, and full mucosal healing was observed by day 14. The patient was discharged with residual hyperpigmentation and referred for diabetes management. At 30-day follow-up, he exhibited no ocular, renal, or cutaneous (Figures 6-8) sequelae, and SCORTEN reassessment dropped to 0. Informed consent was obtained, and patient anonymity was preserved.

## Discussion

This case underscores the importance of early interventions in SJS/TEN, a life-threatening dermatologic emergency [10]. Drug causality was assessed using the ALDEN algorithm, as detailed in the case presentation section, supporting a probable association with dicloxacillin, a penicillin-class antibiotic [17].

The patient’s survival despite a baseline SCORTEN of 4 (predicted mortality 58%) highlights prognostic complexities and the need for protocols tailored to local contexts [8]. Although SCORTEN remains the prognostic gold standard [3], its accuracy varies among different ethnic populations. For instance, Indian cohorts show SCORTEN overestimates mortality in low-risk groups (scores 0-1) and underestimates risk in high-score patients (≥3) [8]. Similarly, Brazilian cohorts demonstrate overprediction of mortality in immunocompetent patients but underperformance in those with advanced HIV, emphasizing the need for context-specific validation [9]. Recent US data report age- and sex-adjusted inhospital mortality rates of 4.8% for SJS, 19.4% for SJS/TEN overlap, and 14.8% for TEN. Hematological malignancies (non-Hodgkin’s lymphoma: OR = 5.25; leukemia: OR = 2.19), septicemia (OR = 7.80), and renal failure (OR = 3.92) emerged as independent mortality predictors, underscoring the impact of comorbidities and specific ethnic groups in refining risk stratification beyond SCORTEN [10].

To our knowledge, no formally documented SJS/TEN overlap cases have been reported in Nicaragua, aside from a 2007 medical thesis (undergraduate thesis: Cálix Martínez et al., Dermatosis producidas por medicamentos en pacientes ingresados al servicio de Medicina Interna del H.E.O.D.R.A Noviembre-Diciembre del 2007, Hospital Escuela Oscar Danilo Rosales Arguello, HEODRA, León, Nicaragua), which lacked sufficient clinical detail to meet current diagnostic criteria. This thesis described a female patient aged 45-59 years with “SJS and erythema multiforme” alongside nephritic syndrome. However, that report lacked critical clinical details, such as the percentage of epidermal detachment, extent of mucosal involvement, or precise timeline, required for a contemporary diagnosis of SJS/TEN overlap (10%-30% BSA detachment) [1]. In contrast, our case explicitly fulfills these criteria, with 30% BSA detachment and extensive mucosal compromise, representing the first well-documented and explicitly classified SJS/TEN overlap syndrome in Nicaragua according to current diagnostic standards [1]. The absence of a national registry or epidemiological data further underscores a significant gap in the recognition, reporting, and surveillance of this life-threatening condition in our setting, highlighting the importance of case documentation in stimulating awareness and research.

The etiological spectrum of SJS/TEN typically involves antibiotics, anticonvulsants, and analgesics as the most implicated drug classes, with sulfonamides, phenytoin, and allopurinol being frequent culprits [12]. In this case, dicloxacillin, a penicillin derivative, was implicated. While HLA allele screening, such as HLA-B58:01 for allopurinol or HLA-B15:02 for carbamazepine, has demonstrated utility in identifying patients at high risk for severe cutaneous adverse reactions [11], its relevance varies depending on the culprit drug. Currently, no well-established HLA associations support routine screening for penicillin-induced SJS/TEN. Moreover, HLA typing was unavailable at our hospital during the acute phase, reflecting limited access to pharmacogenetic testing in our setting and emphasizing the need to develop preventive strategies appropriate for resource-limited contexts.

Management of SJS/TEN centers on prompt withdrawal of the offending drug, supportive care, and immunomodulatory therapy [6,7]. The role of systemic corticosteroids remains debated. A 2022 Cochrane meta-analysis found no clear mortality benefit with corticosteroids (risk ratio, RR: 2.55; 95% confidence interval, CI, 0.72-9.03; very low-certainty evidence), with important outcomes like time to reepithelialization and length of hospital stay often unreported [6]. Similarly, intravenous immunoglobulin (IVIG) and cyclosporine showed inconclusive mortality benefits due to low-quality evidence, though cyclosporine suggested potential mortality reduction compared to IVIG (RR: 0.13; 95% CI: 0.02-0.98) [6]. Etanercept, a tumor necrosis factor-alpha inhibitor, may reduce mortality compared to corticosteroids; however, the confidence intervals include both benefits and harms, necessitating further research [6].

In contrast, several smaller observational studies have reported beneficial effects of corticosteroids. Araki et al. (n = 5) demonstrated that high-dose methylprednisolone preserved corneal stem cells and vision [14]. Kardaun and Jonkman (n = 12) observed an 8.3% mortality rate versus SCORTEN-predicted 25% with dexamethasone use [15]. Hirahara et al. (n = 8) reported 100% survival with pulse corticosteroids and reduced Interferon-gamma-γ/interleukin-6 levels [4]. Ye et al. (n = 56) found actual mortality of 1.8% vs. 15% predicted with moderate-to-high-dose glucocorticoids, and only mild complications [5]. The Indian guidelines recommend systemic corticosteroids within the first 72 hours of onset (level II evidence, grade B recommendation) [16].

In our case, corticosteroids were used cautiously due to the lack of availability of alternatives such as IVIG or cyclosporine. Notably, no secondary infections or corticosteroid-associated complications were observed, consistent with some reports supporting early corticosteroid use under close monitoring [18]. The short treatment duration and rigorous clinical surveillance likely contributed to this favorable safety profile.

Finally, the patient’s survival despite severe presentation highlights the importance of comprehensive supportive care and multidisciplinary management, though conclusions from single cases are limited. The discrepancy between predicted and observed mortality also suggests that integrating comorbidities, ethnic-specific factors, and new biomarkers could enhance risk stratification beyond SCORTEN, particularly in resource-limited settings [14,15,19]. The paucity of epidemiological data in Nicaragua, including the lack of a formal registry and the scarcity and incompleteness of prior reports, such as the HEODRA thesis, calls for enhanced surveillance and research to improve understanding, prevention, and management of SJS/TEN in this region.

## Conclusions

This case represents the first documented instance of SJS/TEN overlap syndrome reported in Nicaragua, contributing valuable data to the limited regional literature on severe drug-induced cutaneous reactions. The patient’s survival following early administration of mild-dose dexamethasone alongside supportive care illustrates a potential treatment approach feasible in resource-limited settings. However, as this is a single case report without controls, definitive conclusions about the efficacy of corticosteroids or treatment protocols cannot be drawn. The absence of pharmacogenomic testing reflects current limitations in resource availability in Central America, highlighting the need to improve access to such tools for personalized prevention and risk assessment of drug hypersensitivity reactions. Future multicenter studies and collaborations are necessary to better understand genetic predispositions in mestizo populations and to evaluate the applicability and calibration of prognostic tools such as SCORTEN in Latin American contexts.
